# Supplementary material for: Tunable circular dichroism through absorption in coupled optical modes of twisted triskelia nanostructures
Source: Sci Rep. 2022 Jan 7;12:26. doi: 10.1038/s41598-021-03908-2 (PMC8742006; doi:10.1038/s41598-021-03908-2)
Supplement: Supplementary file 1 — Supplementary Figures. [file 41598_2021_3908_MOESM1_ESM.pdf]

Supplementary Information

Tunable Circular Dichroism through Absorption in Coupled Optical Modes of Twisted Triskelia Nanostructures

Javier Rodríguez-Álvarez*, Antonio García-Martín, Arantxa Fraile Rodríguez, Xavier Batlle and Amílcar Labarta

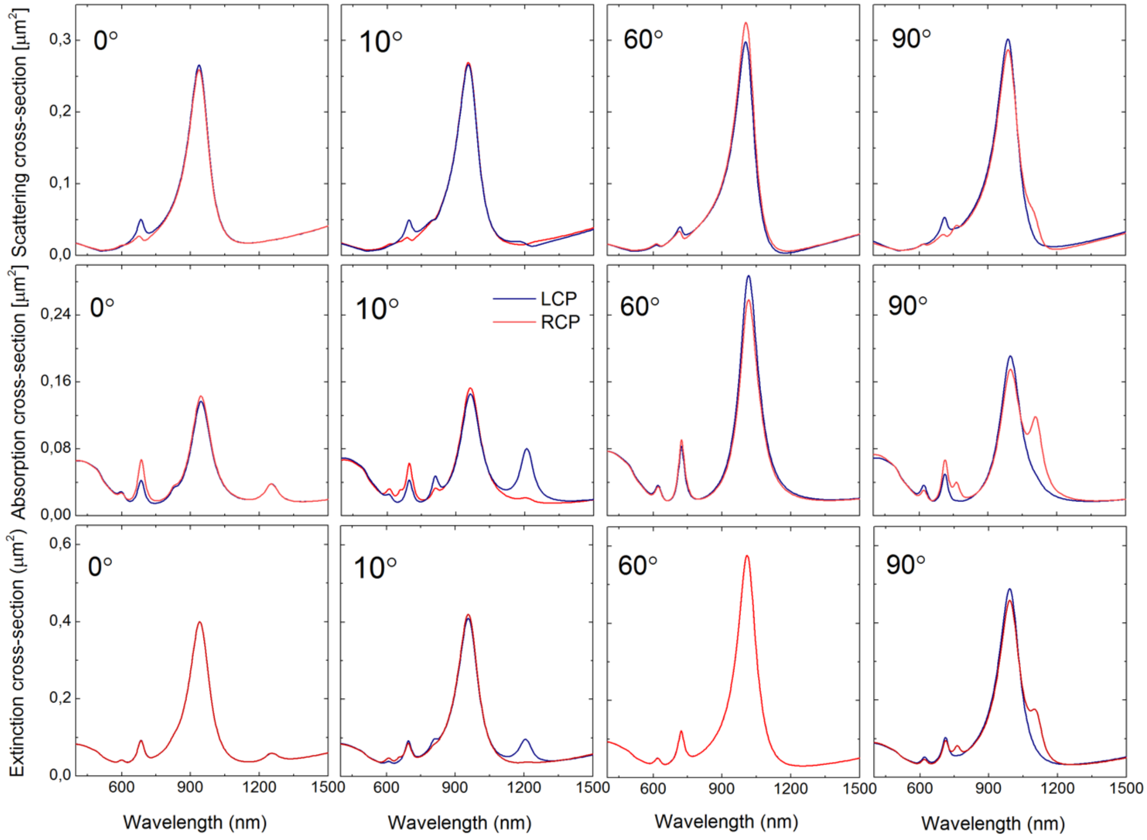

Figure S1. Absorption, scattering and extinction cross-sections under LCP (blue solid line) and RCP (red solid line) light for a double triskelia system forming an anticlockwise in-plane angle of 0°, 10°, 60° and 90° and at an edge-to-edge distance $d = 30$ nm (see Fig. 1(b)).

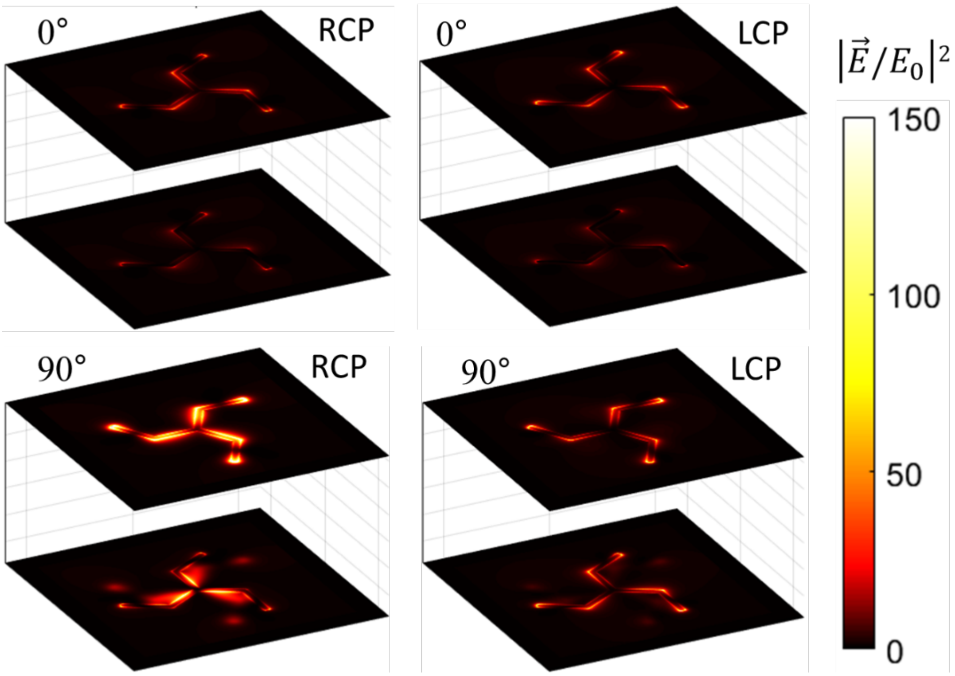

Figure S2. Near-field distributions of the square modulus of the electric field normalized to that of the incident light under RCP and LCP illumination for in-plane angles of 0° and 90°, at a wavelength of 1100 nm.

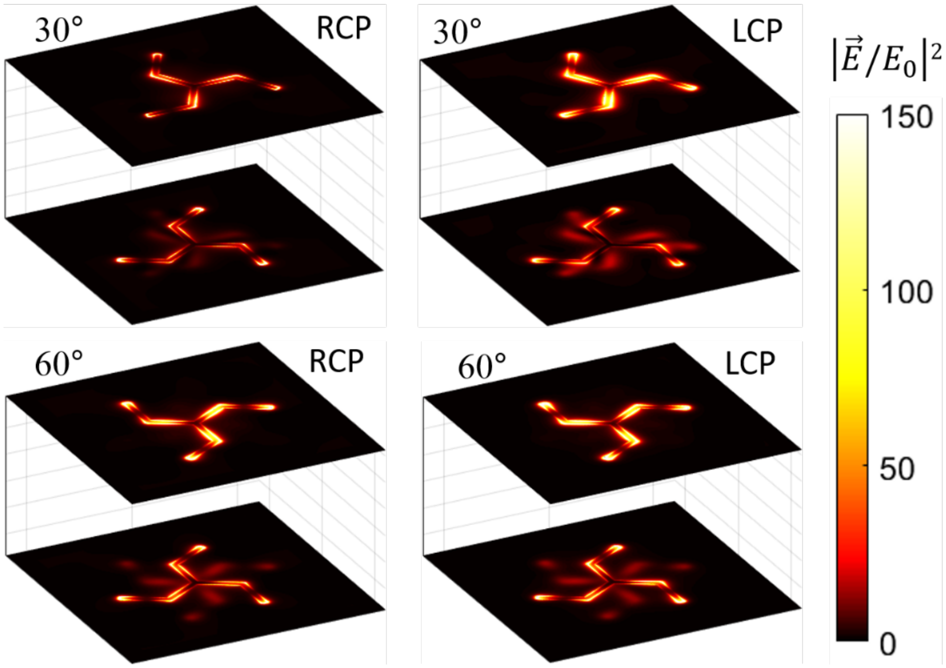

Figure S3. Near-field distributions of the square modulus of the electric field normalized to that of the incident light under RCP and LCP illumination for in-plane angles of 30° and 60°, at a wavelength of 1000 nm.

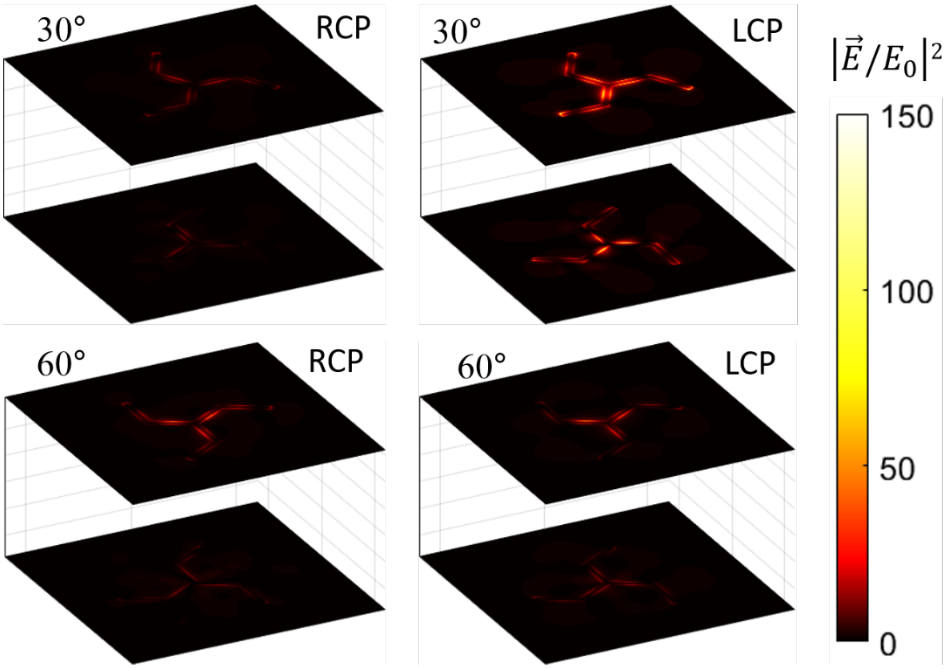

Figure S4. Near-field distributions of the square modulus of the electric field normalized to that of the incident light under RCP and LCP illumination for in-plane angles of 30° and 60°, at a wavelength of 760 nm.

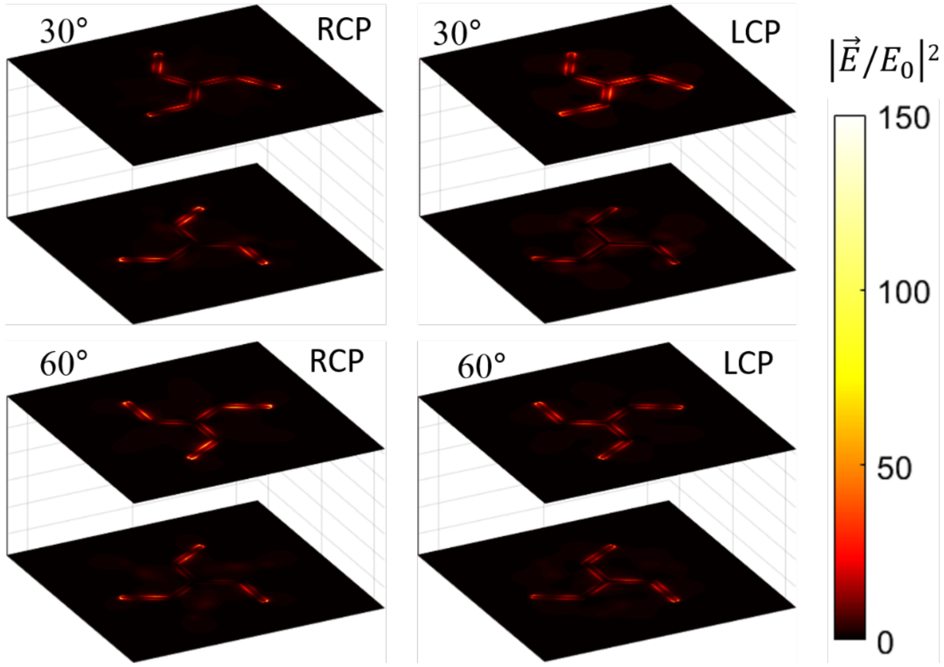

Figure S5. Near-field distributions of the square modulus of the electric field normalized to that of the incident light under RCP and LCP illumination for in-plane angles of 30° and 60°, at a wavelength of 710 nm.
